# Safety, pharmacokinetics, and pharmacodynamics of intravenous ferric carboxymaltose in children with iron deficiency anemia

**DOI:** 10.1038/s41390-023-02644-9

**Published:** 2023-05-19

**Authors:** Bartosz Korczowski, Colm Farrell, Mark Falone, Nicole Blackman, Trudy Rodgers

**Affiliations:** 1 Department of Pediatrics, Institute of Medical Sciences, Medical College, University of Rzeszów, Rzeszów, Poland; 2 ICON plc, Reading, Buckinghamshire UK; 3 American Regent, Inc., Shirley, NY USA

## Abstract

**Background:**

Iron deficiency is the primary cause of anemia in children. Intravenous (IV) iron formulations circumvent malabsorption and rapidly restore hemoglobin.

**Methods:**

This Phase 2, non-randomized, multicenter study characterized the safety profile and determined appropriate dosing of ferric carboxymaltose (FCM) in children with iron deficiency anemia. Patients aged 1–17 years with hemoglobin <11 g/dL and transferrin saturation <20% received single IV doses of undiluted FCM 7.5 mg/kg (*n* = 16) or 15 mg/kg (*n* = 19).

**Results:**

The most common drug-related treatment-emergent adverse event was urticaria (in three recipients of FCM 15 mg/kg). Systemic exposure to iron increased in a dose-proportional manner with approximate doubling of mean baseline-corrected maximum serum iron concentration (157 µg/mL with FCM 7.5 mg/kg; and 310 µg/mL with FCM 15 mg/kg) and area under the serum concentration–time curve (1901 and 4851 h·µg/mL, respectively). Baseline hemoglobin was 9.2 and 9.5 g/dL in the FCM 7.5 and 15 mg/kg groups, respectively, with mean maximum changes in hemoglobin of 2.2 and 3.0 g/dL, respectively.

**Conclusions:**

In conclusion, FCM was well tolerated by pediatric patients. Improvements in hemoglobin were greater with the higher dose, supporting use of the FCM 15 mg/kg dose in pediatric patients (Clinicaltrials.gov NCT02410213).

**Impact:**

- This study provided information on the pharmacokinetics and safety of intravenous ferric carboxymaltose for treatment of iron deficiency anemia in children and adolescents.
- In children aged 1–17 years with iron deficiency anemia, single intravenous doses of ferric carboxymaltose 7.5 or 15 mg/kg increased systemic exposure to iron in a dose-proportional manner, with clinically meaningful increases in hemoglobin.
- The most common drug-related treatment-emergent adverse event was urticaria.
- The findings suggest that iron deficiency anemia in children can be corrected with a single intravenous dose of ferric carboxymaltose and support use of a 15 mg/kg dose.

## Introduction

Iron deficiency is the world’s most common nutritional deficiency affecting children, the primary cause of anemia globally, and an important factor contributing to ongoing disability.^1–3^ The global prevalence of anemia is approximately 33%,^3^ with the highest burden in children aged <5 years, among whom approximately 40% are iron deficient.^4^ In the United States, the prevalence of iron deficiency anemia (IDA) in children aged 1–3 years ranges from 0.9% to 4.4% and varies by race/ethnicity and socioeconomic status.^5^ IDA is associated with poor outcomes in young children, including impaired neurocognitive development and motor and behavioral effects that may persist after the iron deficiency has been corrected and the anemia has resolved.^6,7^

Correction of IDA in pediatric patients involves iron supplementation, which may be administered by oral or parenteral routes.^2,8^ Oral iron supplements are convenient and inexpensive; however, poor tolerability attributed to nausea, vomiting, constipation, and metallic taste is a frequent cause of nonadherence and treatment failure.^8–10^ Although oral iron therapy is usually effective, parenteral iron preparations should be used when malabsorption is present or when gastrointestinal side effects result in noncompliance.

Intravenous (IV) iron formulations circumvent malabsorption, facilitate rapid repletion of iron stores and hemoglobin levels, and have the potential to correct anemia after a single dose.^8,11^ The use of parenteral iron supplementation has historically been limited to selected patients because of concerns over potentially life-threatening hypersensitivity reactions due to high-molecular-weight (HMW) dextran, which was a component of early formulations.^8,12^ Because of safety concerns, use of parenteral iron products containing HMW dextran is not recommended, and these products have been removed from Western European and US markets.^13,14^

Newer formulations that do not contain HMW dextran are much safer, with estimated serious adverse events rates of <1:250,000 administrations,^15,16^ and thus have the potential to modify clinical practice.^8,12^ Six such iron formulations are available worldwide for clinical use in adults (ferric gluconate, iron sucrose, low-molecular-weight iron dextran, ferric carboxymaltose [FCM], ferric derisomaltose, and ferumoxytol).^14^

A substantial proportion of the pediatric literature on the treatment of severe anemia is derived from studies in children with chronic kidney disease (CKD).^17^ For this reason, there is insufficient data on the safety and efficacy of IV iron preparations when used to treat a broader range of anemias, including nutritional iron deficiency, in children and adolescents, which limits treatment options for this population. For example, in pediatric patients 2 years of age or older, iron sucrose is only approved for use to treat IDA in patients with CKD and has not been studied in patients younger than 2 years of age.^18^

The most commonly used IV iron preparation in pediatric patients, iron sucrose, has a recommended maximum single dose for iron maintenance treatment that should not exceed 100 mg and should be infused over 5 to 60 min in pediatric patients with CKD who are receiving hemodialysis or erythropoietin therapy, with no established dosing for iron replacement treatment.^18^ In real-world experience, iron sucrose has been used to treat IDA of multiple etiologies in pediatric patients, but often requiring multiple doses and with total dose infusion not possible.^9,17,19^ Thus, multiple clinic visits or one prolonged hospital stay may be required to restore iron levels in a child with, for example, nutritional anemia. The availability of IV iron formulations that allow for more rapid administration of higher doses would be more convenient and efficient for the patient and the healthcare system.

The labels for several newer iron preparations allow for the administration of higher doses of iron in adults, for example, ferumoxytol (510 mg over at least 15 min),^20^ FCM (750 mg over at least 15 minutes),^21^ or ferric derisomaltose (1000 mg over at least 20 min).^22^ The dose may need to be repeated several days later for administration of the total required dose of iron. Until recently, none of these products were approved for use in children.

Studies of the safety, effectiveness, and optimal dosing of newer iron products in pediatric patients are required in order to allow access to these products. FCM is a stable Type I polynuclear iron (III) hydroxide carbohydrate complex that was developed as an IV iron replacement therapy and is widely available for the treatment of IDA.^12,21,23^ In the US, FCM is indicated for IDA in adult patients who have either an intolerance to or an unsatisfactory response to oral iron supplements or who have non-dialysis dependent CKD, and was recently approved for use in pediatric patients aged ≥1 year who have either an intolerance to or an unsatisfactory response to oral iron.^21^ FCM has been shown to be safe and effective for the treatment of IDA in adults,^24–27^ including during pregnancy and post-partum, as well as in patients with irritable bowel disease, abnormal uterine bleeding, chronic heart failure, pulmonary arterial hypertension, and CKD.^27–29^ A pooled analysis of 10 randomized trials of FCM in IDA has shown that FCM, even when administered at high doses, has a tolerability profile that is comparable to or better than those of other parenteral iron preparations.^30^ Given the unmet need for safe, well-tolerated, and rapidly effective treatments for IDA in pediatric patients, we initiated a clinical trial to evaluate FCM in pediatric patients with IDA.

The objectives of this Phase 2 study were to characterize the safety profile, pharmacokinetics (PK), and pharmacodynamics (PD) of single parenteral doses of FCM and to determine appropriate dosing in pediatric patients with IDA.

## Methods

This Phase 2, open-label, non-randomized, multicenter, dose-finding study (clinicaltrials.gov NCT02410213) was conducted at eight sites in Poland and two in Russia; sites were either large hospitals or a specialist outpatient clinic.

### Ethics

The study was conducted in accordance with the Declaration of Helsinki, all applicable local and state regulations, and International Council for Harmonisation guidelines. The protocol, amendments to the protocol, and the informed consent form were reviewed and approved by institutional review boards at each study site prior to initiation of the study. Ethical approval for the eight study sites in Poland was provided by the Bioethics Committee at the Medical University in Lublin, Lublin, Poland. For the two study sites in the Russian Federation, ethical approval was provided by the Ethics Committee at State Budgetary Educational Institution of Higher Professional Education (SBEI HPE), Ryazan State Medical University named after academician I.P. Pavlov, Ministry of Public Health of the Russian Federation, Ryazan, Russian Federation or the Ethics Committee at SBEI HPE, Saint-Petersburg State Pediatric Medical University, Ministry of Healthcare of the Russian Federation, St Petersburg, Russian Federation.

Informed consent was obtained from all participants, and a written consent form was signed by the participant and/or their legal representative in their native language prior to study participation. A Data Safety Monitoring Board (DSMB) provided oversight.

### Patients

Boys and girls eligible for enrollment at study sites in Poland were 1–17 years of age and those in Russia were 6–17 years of age. At screening, eligible patients had a hemoglobin concentration <11 g/dL and a transferrin saturation (TSAT) <20%. Therapy with an erythropoietin-stimulating agent was permitted, provided that the dose had been stable for more than 8 weeks prior to screening and that no changes in the dose or product were anticipated for the duration of the trial.

Patients were excluded with a ferritin level >300 ng/mL at screening, with a body mass index (BMI) ≤5th percentile for age, or, for individuals who were enrolled while 1 year of age, with body weight <12 kg. Other exclusion criteria included active infection; anemia for reasons other than iron deficiency; receipt of immunosuppressive therapy, other than steroid therapy, that could exacerbate anemia; receipt of IV iron and/or a blood transfusion within 4 weeks prior to screening; history of acquired iron overload, hemochromatosis, or other iron accumulation disorder; severe diseases of the liver or the hematopoietic or cardiovascular system; CKD treated with hemodialysis; and/or evidence of infection with human immunodeficiency virus, or hepatitis B or C viruses with evidence of active hepatitis.

### Study design and treatment

Patients who met the selection criteria entered a ≤14-day screening period to assess eligibility. Eligible patients were enrolled sequentially in one of two dose groups in which they received a single IV dose of FCM (Vifor Pharma Ltd., St. Gallen, Switzerland) (either 7.5 mg/kg or 15 mg/kg, with a maximum total dose of 750 mg). Enrollment in the group to receive the higher dose of FCM (15 mg/kg) began after all patients in the lower dose group (FCM 7.5 mg/kg) had received study drug and been observed for 4 weeks post-dose with no safety concerns as determined by the DSMB on the basis of an interim analysis of the FCM 7.5 mg/kg cohort.

FCM was administered as an undiluted solution at a rate of 100 mg/min. Doses less than 100 mg were administered as a slow undiluted IV push injection within 1 min. All patients were followed for 35 days. After infusion of FCM on Day 0, patients returned to the clinic at 24, 48, and 72 h post-dose, and subsequently on study Days 14, 28, and 35 for safety, PK, and PD assessments.

### Safety assessments

Safety was assessed by the incidence of treatment-emergent adverse events (TEAEs), serious TEAEs, and mean changes from baseline in clinical laboratory values at each scheduled visit. Blood for clinical laboratory values was collected at screening (up to Day −14), at 72 h post-dose, and on Days 14, 28, and 35. Medical Dictionary for Regulatory Activities (MedDRA) version 17.0 was used to classify all TEAEs by system organ class and preferred term; severity was classified with National Cancer Institute Common Terminology Criteria for Adverse Events version 4.0.

### Pharmacokinetic and pharmacodynamic assessments

Blood samples were collected on Day −1 at 8 AM, 12 PM, and 8 PM to characterize each patient’s baseline iron status, pre-dose on Day 0, and then at 1, 2, 6, 12, 24, 48, and 72 h post-dose. Whole blood samples were collected in 5 mL evacuated red-top blood collection tubes (no anticoagulant) and were allowed to clot while standing at room temperatures and then centrifuged at 2000g. Serum samples (minimum volume 800 µL) were then transferred to cryovials, frozen at –20 °C and shipped on dry ice for analysis (Butterworth Laboratories Limited, Teddington, Middlesex, UK). Serum samples were analyzed for iron content using inductively coupled plasma-mass spectrometry. The assay was validated over the range of 1–1000 µg/mL and does not distinguish between endogenous serum iron and that derived from the FCM infusion.

PK parameters were determined for each patient. The primary PK parameters for iron were maximum serum concentration (*C*_max_), time to *C*_max_ (*T*_max_), area under the serum concentration-time curve (AUC) from time zero to the last sampling time with a quantifiable concentration (AUC_0-last_), AUC from time zero extrapolated to infinity (AUC_0-inf_), and elimination half-life (*t*_½_), with baseline values subtracted from all measured samples.

Secondary PK parameters for iron were mean residence time, apparent serum clearance (CL), and apparent volume of distribution (*V*_d_), where *V*_d_ refers to the apparent volume of distribution associated with the terminal phase calculated as dose of iron in mg/(*λ*_*z*_ × AUC_0-inf_).

PK analyses were conducted by ICON plc (Reading, UK) using Phoenix WinNonlin v6.3 (Certara Corp, St Louis, MO). PK parameters for baseline-corrected total serum iron were calculated using noncompartmental analysis (WinNonlin Model 200–202 for IV dosing). Actual elapsed time from the start of the infusion and actual dose amounts were used in calculations. Concentrations determined to be below the limit of quantitation were assigned a value of zero before achievement of *C*_max_ and were treated as missing values after achievement of *C*_max_.

PD assessments included serum ferritin, serum transferrin, serum hemoglobin, reticulocyte count, and TSAT. Blood samples for all PD assessments were collected at screening (up to 14 days before treatment Day 0), at 72 h post-dose, and on Days 14, 28, and 35. The change from baseline in ferritin, hemoglobin, and TSAT at each scheduled visit; and the change from baseline to the highest post-dose value in serum ferritin, transferrin, hemoglobin, reticulocyte count, and TSAT were summarized.

### Statistical considerations

Sample size determination followed practical, feasibility, and empirical considerations for a Phase 2 dose-finding study. The planned enrollment was 32 patients with 16 patients in each treatment group equally distributed by age (eight patients aged 1–6 years and eight aged >6–17 years).

The safety population included all patients who received FCM; the PK population included all patients in the safety population who had at least one measurable concentration of FCM; the PD population included all patients in the safety population who had at least one PD assessment. Baseline was defined as Day 0; if data on Data 0 were missing or not captured, the screening value was used.

No hypothesis testing was planned for this study; only descriptive, summary statistics were planned for assessment of dosing and safety. Statistical programming and analyses were performed using SAS v9.1.3 (SAS Institute Inc., Cary, NC).

## Results

A total of 35 patients with IDA were enrolled between February 19, 2015, and January 22, 2017, and treated with FCM 7.5 mg/kg (*n* = 16) or, after the DSMB recommended that the study continue as designed, with FCM 15 mg/kg (*n* = 19) (Supplemental Fig. 1). All 35 patients completed the study. The safety, PK, and PD populations each included 35 patients. However, two patients from the FCM 7.5 mg/kg group were excluded from PK-related summaries (one patient because of missing values and one patient for anomalous values). The median age of patients was 9.8 years (range 1.5–16.9 years) and 12.4 years (range 1.6–17.5 years) in the FCM 7.5 mg/kg and FCM 15 mg/kg groups, respectively (Table 1). A total of 10 of 16 patients (62.5%) treated with FCM 7.5 mg/kg and 9 of 19 patients (47.4%) treated with FCM 15 mg/kg were female. The median BMI in both dose groups was 18.5 kg/m^2^. In the FCM 7.5 mg/kg dose group, IDA due to gastrointestinal disorders (56.3%), insufficient dietary intake (31.3%), and hemophilia (6.3%) were the primary etiologies. In the FCM 15 mg/kg dose group, IDA due to gastrointestinal disorders (57.9%), insufficient dietary intake (26.3%), and heavy uterine bleeding (15.8%) were the primary etiologies. No patients were receiving erythropoietin at screening.

**Table 1:** Demographic and baseline characteristics in pediatric patients with iron deficiency anemia treated with FCM (safety population).

Characteristic	FCM 7.5 mg/kg^a^ (*N* = 16)	FCM 15 mg/kg^a^ (*N* = 19)
Age, years		
Mean ± SD	9.1 ± 6.13	10.3 ± 5.77
Median (range)	9.8 (1.5–16.9)	12.4 (1.6–17.5)
Female sex, *n* (%)	10 (62.5)	9 (47.4)
Weight, kg, median (range)	32.35 (12.1–64.0)	42.0 (13.1–74.0)
Body mass index, kg/m^2^, median (range)	18.5 (13.4–22.7)	18.5 (13.6–25.9)
Body surface area, m^2^, median (range)	0.87 (0.55–1.73)	1.36 (0.56–1.91)
Iron deficiency anemia, *n* (%)	16 (100)	19 (100)
Primary cause of iron deficiency anemia, *n* (%)		
Gastrointestinal disorders^b^	9 (56.3)	11 (57.9)^c^
Insufficient dietary iron intake	5 (31.3)	5 (26.3)^c^
Heavy uterine bleeding	0 (0)	3 (15.8)
Hemophilia	1 (6.3)	0 (0)
Unknown	1 (6.3)	2 (10.5)

*FCM* ferric carboxymaltose, *SD* standard deviation.

^a^Maximum 750 mg.

^b^Gastrointestinal disorders included: celiac disease; gastritis/*Helicobacter pylori* infection; gastrointestinal bleeding/esophageal varices/gastroesophageal reflux disease; inflammatory bowel disease/Crohn’s disease; malabsorption.

^c^Two patients had both gastrointestinal disorder (gastritis) and insufficient dietary iron intake.

### Safety

A total of 9 of 16 patients (56.3%) who received FCM 7.5 mg/kg and 12 of 19 patients (63.2%) who received FCM 15 mg/kg experienced at least 1 TEAE (Table 2). The most common TEAEs were pyrexia (12.5%) and rash (12.5%) in patients who received FCM 7.5 mg/kg, and rhinorrhea (15.8%), urticaria (15.8%), hyperthermia (10.5%), and upper respiratory tract infection (10.5%) in patients who received FCM 15 mg/kg (Table 2). Two patients who received FCM 7.5 mg/kg experienced a serious TEAE (one each upper respiratory tract infection and sinusitis, neither of which were considered to be related to the study drug). Most patients who had TEAEs had TEAEs of Grade 1 or 2 in severity; the only patient who had a severe TEAE received FCM 7.5 mg/kg and experienced sinusitis (Grade 3) that was not considered related to the study drug. Mild and transient hypophosphatemia occurred in the FCM 15 mg/kg group (Supplemental Table 1). Mean phosphate levels decreased in both cohorts at 72 h post-dose but returned to baseline or near-baseline levels by Day 14 in the FCM 7.5 mg/kg group and by Day 28 in the FCM 15 mg/kg group.

**Table 2:** Overview of treatment-emergent adverse events (TEAEs) in pediatric patients with iron deficiency anemia treated with FCM (safety population).

TEAE	FCM 7.5 mg/kg^a^ (*N* = 16)	FCM 15 mg/kg^a^ (*N* = 19)
Any TEAE	9 (56.3)	12 (63.2)
≥1 serious TEAE	2 (12.5)	0
≥1 severe TEAE^b^	1 (6.3)	0
≥1 study drug-related TEAE^c^	3 (18.8)	6 (31.6)
TEAEs occurring in ≥2 patients in either group		
Rhinorrhea	0	3 (15.8)
Urticaria	0	3 (15.8)
Pyrexia	2 (12.5)	0
Hyperthermia	0	2 (10.5)
Rash	2 (12.5)	1 (5.3)
Upper respiratory tract infection	1 (6.3)	2 (10.5)

Note: Data are presented as *n* (%).

*FCM* ferric carboxymaltose, *TEAE* treatment-emergent adverse event.

^a^Maximum 750 mg.

^b^Common Terminology Criteria for Adverse Event Grade 3, 4, or 5.

^c^Possibly or probably related to the study drug.

A total of three patients (18.8%) who received FCM 7.5 mg/kg and six patients (31.6%) who received FCM 15 mg/kg experienced study drug-related TEAEs. The most common study drug-related TEAE was urticaria, which was reported by three patients who received FCM 15 mg/kg (none who received FCM 7.5 mg/kg) after infusion on Day 0. Other study drug-related TEAEs, all of which occurred in one patient each, included infusion site pruritus, thirst, and hot flush in patients who received FCM 7.5 mg/kg and upper abdominal pain, gastroduodenitis, hyperthermia, injection site pain, increased alanine aminotransferase levels, headache, pruritus, rash, and hypertension in patients who received FCM 15 mg/kg. No consistent dose-response relationships across age groups in changes in blood pressure or heart rate were observed.

### Pharmacokinetics

**Fig. 1:**
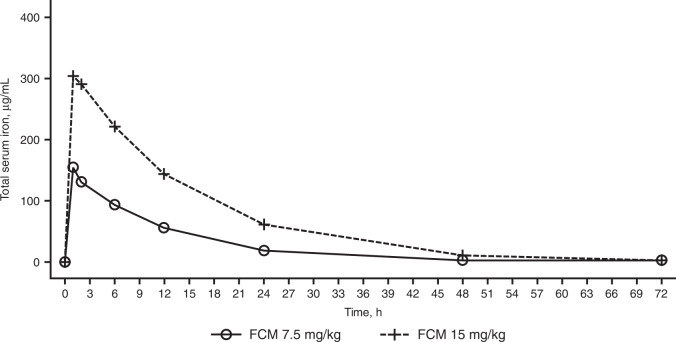
Mean baseline-corrected total serum iron concentration–time profiles in pediatric patients treated with FCM (PK population). Solid line indicates FCM 7.5 mg/kg (*n* = 15)^a^. Dashed line indicates FCM 15 mg/kg (*n* = 19). ^a^One patient was excluded from the PK analysis set (*N* = 16) for the FCM 7.5 mg/kg group for all time points because of anomalous and consistently high concentrations; *n* = 15 for time points other than 1 and 2 h post dose; *n* = 14 for 1 and 2 h post dose because of missing assessments at those time points for one patient. *FCM* ferric carboxymaltose, *PK* pharmacokinetics.

Systemic exposure to iron increased in a dose-proportional manner (Fig. 1). Median *T*_max_ occurred at approximately 1 h after infusion of FCM in both dose groups (Table 3 and Fig. 1). Mean baseline-corrected *C*_max_ for total serum iron in patients who received FCM 15 mg/kg was approximately twice that of patients who received FCM 7.5 mg/kg: 310 versus 157 µg/mL, respectively **(**Table 3). Systemic exposure in the group that received FCM 15 mg/kg was more than double that in the group that received FCM 7.5 mg/kg as indicated by values for AUC_0-last_ (4851 versus 1901 h·µg/mL, respectively) and AUC_0-inf_ (4906 versus 1939 h·µg/mL, respectively) (Table 3). The mean *t*_½_, *V*_d_, and CL were similar in both groups after single 7.5 and 15 mg/kg doses of FCM (Table 3). Dose-normalized values for *C*_max_, AUC_0-last_, and AUC_0-inf_ for the two dose groups are presented in Table 4.

**Table 3:** Baseline-corrected total serum iron pharmacokinetic parameters in pediatric patients with iron deficiency anemia after treatment with FCM (PK population).

PK parameter	FCM 7.5 mg/kg^a^ (*N* = 14)^b^	FCM 15 mg/kg^a^ (*N* = 19)
*T*_max_, h	1.03 (0.98–1.98)	1.12 (1.03–2.12)
*C*_max_, µg/mL	157 (53.07)	310 (42.9)
AUC_0-last_, h·µg/mL	1901 (639.1)	4851 (1046.3)
AUC_0-inf_, h·µg/mL	1939 (656.6)	4906 (1045.2)
*t*_½_, h	9.76 (2.338)	9.84 (1.996)
*V*_d_, mL	1798 (993.2)	1510 (850.6)
CL, mL/min	2.05 (0.9)	1.70 (0.8)

Note: Data are presented as arithmetic means (SD), with the exception of *T*_max_, for which the median (range) is presented.

*AUC*_*0-last*_ area under the serum concentration–time curve from time zero to the last sampling time with a quantifiable concentration, *AUC*_*0-inf*_ extrapolated area under the serum concentration–time curve from time zero to infinity, *CL* apparent serum clearance, *C*_*max*_ maximum serum concentration, *FCM* ferric carboxymaltose, *PK* pharmacokinetic, *SD* standard deviation, *t*_*½*_ half-life, *T*_*max*_ time to *C*_max_, *V*_*d*_ apparent volume of distribution.

^a^Maximum 750 mg.

^b^Two patients were excluded from the PK analysis set (*N* = 16); one patient had anomalous and consistently high concentrations of serum iron, and one patient had an incomplete profile.

**Table 4:** Dose-normalized baseline-corrected total serum iron PK parameters in pediatric patients with iron deficiency anemia after treatment with FCM (PK analysis set).

PK parameter^a^	FCM 7.5 mg/kg^b^ (*N* = 14)^c^	FCM 15 mg/kg^b^ (*N* = 19)
*C*_max_/dose, µg/mL/mg	0.88 (0.50)	0.84 (0.53)
AUC_0-last_/dose, h·µg/mL/mg	9.90 (4.80)	11.92 (5.63)
AUC_0-inf_/dose, h·µg/mL/mg	10.09 (4.85)	12.07 (5.70)

Note: Data are presented as arithmetic means (SD).

*AUC*_*0-last*_ area under the serum concentration–time curve from time zero to the last sampling time with a quantifiable concentration, *AUC*_*0-inf*_ extrapolated area under the serum concentration–time curve from time zero to infinity, *C*_*max*_ maximum serum concentration, *FCM* ferric carboxymaltose, *PK* pharmacokinetic, *SD* standard deviation.

^a^Total dose administered in mg was used in calculations.

^b^Maximum 750 mg.

^c^Two patients were excluded from the PK analysis set (*N* = 16); one patient had anomalous and consistently high concentrations of serum iron, and one patient had an incomplete profile.

### Pharmacodynamics

After treatment with FCM, consistent increases in serum ferritin, hemoglobin, reticulocyte count, and TSAT and consistent decreases in transferrin were observed compared with baseline (Table 5). Baseline hemoglobin concentration was 9.2 g/dL in the FCM 7.5 mg/kg group and 9.5 g/dL in the FCM 15 mg/kg group, and the mean maximum change in hemoglobin concentration during 35 days of observation post-treatment for each of the dose groups was 2.2 g/dL and 3.0 g/dL, respectively.

**Table 5:** Pharmacodynamic changes in pediatric patients with iron deficiency anemia treated with FCM (PD population).

PD parameter	FCM 7.5 mg/kg^a^ (*N* = 16)		FCM 15 mg/kg^a^ (*N* = 19)	
	Baseline	Maximum change from baseline^b^	Baseline	Maximum change from baseline^b^
Ferritin, ng/mL	8.9 (9.94)	455.9 (513.58)	21.0 (69.24)^c^	575.2 (159.39)^c^
Transferrin, mg/dL	334.5 (71.82)	−18.0 (50.11)	355.7 (61.35)	−47.3 (31.0)
Hemoglobin, g/dL	9.2 (1.20)	2.2 (1.12)	9.5 (0.81)	3.0 (1.03)
Reticulocyte count, %	1.6 (0.71)	1.7 (3.33)	1.4 (0.43)	0.9 (0.85)
TSAT, %	7.5 (4.64)	34.2 (24.51)	3.4 (1.61)	52.1 (27.76)

Note: Data are presented as arithmetic means (SD).

*FCM* ferric carboxymaltose, *PD* pharmacodynamic, *SD* standard deviation, *TSAT* transferrin saturation.

^a^Maximum 750 mg.

^b^Largest magnitude change from baseline, regardless of direction.

^c^*n* = 18.

Mean values from baseline through Day 35 for ferritin, transferrin, hemoglobin, reticulocyte count, and TSAT are shown in Table 6. Mean changes from baseline in serum ferritin and TSAT were greatest on Day 3 after administration of FCM. Mean ferritin concentration at baseline and Day 3 was 8.8 ng/mL and 464.8 ng/mL, respectively, in patients who received FCM 7.5 mg/kg and 21.0 ng/mL and 595.0 ng/mL, respectively, in patients who received FCM 15 mg/kg. Values remained higher in the FCM 15 mg/kg group than in the FCM 7.5 mg/kg group for the remainder of follow-up and on Day 35 were 44.0 ng/mL in the FCM 7.5 mg/kg group and 72.6 ng/mL in the FCM 15 mg/kg group. A similar pattern was observed in TSAT values in that the greatest increase from baseline occurred at Day 3 and values continued to be above baseline at Day 35 in both treatment groups.

**Table 6:** Pharmacodynamic parameters at baseline and to Day 35 in pediatric patients with iron deficiency anemia treated with FCM (PD population).

PD parameter	FCM 7.5 mg/kg^a^ (*N* = 16)	FCM 15 mg/kg^a^ (*N* = 19)
Ferritin, ng/mL		
Baseline	8.9 (9.94)	21.0 (69.24)^b^
Day 3	464.8 (510.95)	595.0 (192.96)
Day 14	72.8 (71.49)	157.8 (201.55)
Day 28	47.2 (85.19)	94.4 (148.31)^b^
Day 35	44.0 (97.14)	72.6 (84.64)
Transferrin, mg/dL		
Baseline	334.5 (71.82)	355.7 (61.35)
Day 3	301.4 (45.29)	301.8 (43.92)
Day 14	275.6 (51.82)	279.3 (50.61)
Day 28	276.5 (62.53)	267.8 (47.24)
Day 35	285.1 (51.97)	264.3 (50.94)
Hemoglobin, g/dL		
Baseline	9.2 (1.20)	9.5 (0.81)
Day 3	9.4 (0.94)	9.6 (1.21)
Day 14	10.6 (0.78)	11.4 (0.87)
Day 28	11.0 (1.11)	12.2 (0.85)
Day 35	11.2 (1.13)	12.3 (0.94)
Reticulocyte count, %		
Baseline	1.6 (0.71)	1.4 (0.43)
Day 3	2.8 (3.67)	1.5 (0.47)
Day 14	1.8 (0.85)	2.2 (0.71)
Day 28	1.2 (0.46)	1.3 (0.33)
Day 35	1.2 (0.85)	1.2 (0.38)
TSAT, %		
Baseline	7.5 (4.64)	3.4 (1.61)
Day 3	41.5 (27.34)	54.9 (28.56)
Day 14	16.6 (13.57)	13.8 (5.86)
Day 28	16.0 (13.47)	16.5 (6.49)^b^
Day 35	17.3 (14.03)	16.9 (7.67)

Note: Data are presented as arithmetic means (SD).

*FCM* ferric carboxymaltose, *PD* pharmacodynamics, *SD* standard deviation, *TSAT* transferrin saturation.

^a^Maximum 750 mg.

^b^*n* = 18.

Mean changes in hemoglobin compared with baseline were small at Day 3; however, by Day 14, the mean hemoglobin concentration had risen from 9.2 g/dL at baseline to 10.6 g/dL in the FCM 7.5 mg/kg cohort and from 9.5 g/dL at baseline to 11.4 g/dL in the FCM 15 mg/kg cohort and continued to increase to Day 35; hemoglobin values remained consistently higher in the FCM 15 mg/kg group than in the FCM 7.5 mg/kg group throughout follow-up. In both treatment groups transferrin concentrations were highest at baseline, decreased by Day 3, and remained below baseline at Day 35. Reticulocyte counts were highest at Day 3 for patients treated with FCM 7.5 mg/kg and at Day 14 for patients treated with FCM 15 mg/kg. At Day 35, reticulocyte counts were below baseline values for both treatment groups.

## Discussion

The results of this Phase 2 trial show that treatment with IV FCM was generally well tolerated and produced clinically meaningful, dose-proportional increases in mean hemoglobin concentrations in pediatric patients with IDA. FCM has very low immunogenic potential, and its properties permit rapid administration (e.g., 15 min) of large, single doses, which allows for rapid repletion of total body iron and supports the utility of FCM in the treatment of IDA in pediatric patients.^28^

The overall nature and incidence of TEAEs and study drug-related TEAEs were similar in the two dose groups. Most TEAEs were Grade 1 or 2 in severity, and no serious TEAE was deemed to be related to the study drug. Urticaria was observed in three FCM recipients in the higher dose group (15 mg/kg) and was the only study drug-related TEAE that occurred in more than one patient. Urticaria may result from a transient hypersensitivity reaction limited to the skin and subcutaneous tissues; however, no consistent dose-response relationship with respect to transient allergic reactions could be established with the small sample size in the present study. A larger series of pediatric patients will have to be monitored to better determine the incidence, severity, and relationship to dose of hypersensitivity reactions to FCM.

This study was completed before FCM was approved by US FDA for the treatment of IDA in pediatric patients aged ≥1 year who have either intolerance to oral iron or an unsatisfactory response to oral iron.^21^ The safety profile of FCM in the present study is consistent with that of a Phase 3 pediatric study conducted in the US in support of this indication (NCT03523117). Among 40 children aged 1 to 17 years with IDA who were randomized to receive FCM 15 mg/kg on Days 0 and 7, 35% experienced an AE.^21^ TEAEs occurring in ≥5% of recipients of FCM were hypophosphatemia, injection site reactions, rash, headache and vomiting.^21^ The incidence of injection site reactions and rash (including urticarial rash) was 8%.^21^ Hypophosphatemia was the most common (13%) AE reported in the Phase 3 pediatric study^21^ and was also observed in adults treated with FCM.^21,26^

The safety profile reported here also aligns with safety results from two single-center retrospective cohort studies of pediatric patients treated with FCM.^31,32^ Among 144 patients aged 18 months to <18 years with IDA or iron deficiency without anemia and poor response to oral therapy treated with a single dose of FCM (maximum dose of 20 mg/kg or 1000 mg total), five patients reported TEAEs potentially related to FCM during a 96-h follow-up period, and no serious TEAEs were reported.^31^ Similarly, in a study of 72 patients aged 9 months to 18 years with IDA refractory to oral iron therapy treated with one or two doses of FCM (15 mg/kg; maximum 750 mg per dose), seven patients reported a TEAE during or immediately after their infusions, with pruritus and/or urticaria as the most common events.^32^

The safety profile in the current study is consistent with that reported in other PK studies with different designs and doses of FCM in adults with IDA.^24–26^ In a dose-escalation study in adults, mild urticaria was reported in two adult patients who received FCM 500 mg infused over 15 min but not in those who received a higher dose in the same study.^26^ No serious treatment-related AEs were reported in three PK studies in adults^24–26^; severe hypophosphatemia occurred in one patient after receiving a single 1000 mg dose.^26^

In previous reports in adults with IDA,^24–26^ single IV doses of FCM produced dose-dependent increases in mean systemic exposure to iron. The geometric mean *t*_½_ observed in children enrolled in the present study (9.5 h with FCM 7.5 mg/kg or 9.6 h with FCM 15 mg/kg) falls within the range of geometric means reported previously in PK studies in adults (7.4–12.3 h),^24–26^ and compares well with the mean *t*_½_ (9.7 h) observed in US children aged 1–17 years after receiving FCM 15 mg/kg (NCT03523117).^21^ Mean systemic exposure in children treated with FCM 15 mg/kg in the present study (AUC_0-last_ 4851 h·µg/mL) was very similar to that in NCT03523117 (AUC_0-72_ 4530 h·µg/mL).^21^

PK data with other IV iron formulations are available for pediatric populations with CKD. Among 49 patients with PK data from a randomized, double-blind study of IV ferric gluconate in iron-deficient pediatric (age ≤15 years) patients undergoing hemodialysis, a single dose of ferric gluconate (1.5 mg/kg or 3.0 mg/kg) resulted in rapid, dose-dependent increases in mean serum iron concentrations (total iron and ferric gluconate-bound iron).^33^ The PK analysis of ferric gluconate-bound iron in the 1.5 mg/kg group demonstrated a mean *t*_½_ of 2.0 h, *C*_max_ of 1287 µg/dL, and AUC_0-inf_ of 9499 h·µg/dL.^33^ In the 3.0 mg/kg group, mean *t*_½_ was 2.5 h, *C*_max_ was 2283 µg/dL, and AUC_0-inf_ was 17,087 h·µg/dL. In a single-dose pharmacokinetic study of iron sucrose, in which 11 patients aged 12–16 years with non-dialysis-dependent CKD received IV bolus doses of iron sucrose 7 mg/kg (maximum 200 mg) administered over 5 min, the *t*_½_ of total serum iron was 8 h, the mean *C*_max_ was 8545 μg/dL, and mean AUC was 31,305 h·µg/mL.^18^

In the current study, FCM resulted in rapid (72 h post-dose), clinically meaningful improvement of PD parameters in pediatric patients with IDA. Maximum mean increases in serum ferritin and TSAT were documented 72 h after administration of a single dose of FCM; the increases from baseline observed in both serum ferritin and TSAT remained consistently higher in patients who received the 15 mg/kg dose than increases in those who received the 7.5 mg/kg dose throughout 4 weeks of follow-up. Treatment with FCM also resulted in rapid and sustained increases in ferritin that were well above the threshold of 10–15 µg/L that is indicative of depleted iron stores in children depending on age.^5,34^

Increases in hemoglobin occurred more slowly and were sustained throughout follow-up in both dose groups. The maximum change in hemoglobin concentration was documented 35 days after administration and was larger in patients who received FCM 15 mg/kg than that in those who received FCM 7.5 mg/kg.

According to World Health Organization criteria, anemia is defined as a hemoglobin concentration <11.0 mg/dL in children aged 6–59 months, <11.5 mg/dL in children aged 5–11 years, and <12.0 mg/dL in children aged 12–14 years.^35^ The hemoglobin levels at baseline (9.2 g/dL and 9.5 g/dL in the FCM 7.5 mg/kg and FCM 15 mg/kg groups, respectively) observed in the present trial correspond to moderate anemia in all three of these age groups.^35^ After a single IV dose of FCM, the mean maximum increases in hemoglobin concentration were observed 35 days post-dose and were 1.9 g/dL and 2.8 g/dL in patients treated with FCM 7.5 mg/kg and FCM 15 mg/kg, respectively. These increases would have been sufficient to normalize the mean hemoglobin level in all age groups in patients receiving FCM 15 mg/kg.

The PD results reported here are generally consistent with previous reports from retrospective cohort studies of pediatric patients treated with FCM. Among pediatric patients with IDA and poor response to oral therapy (*N* = 35–82 with pre- and post-infusion data), a single dose of FCM (maximum 20 mg/kg or 1000 mg total) resulted in mean increases from baseline at 6–12 weeks post-treatment in hemoglobin (22.5 g/L), serum ferritin (69.1 ng/mL), and TSAT (13.1%).^31^ Likewise, pediatric patients with IDA refractory to oral iron therapy treated with one or two doses of FCM (15 mg/kg; maximum 750 mg per dose) and having follow-up testing at 4–12 weeks post-treatment (*N* = 53) showed increases in hemoglobin concentration (from a median of 9.1 g/dL to 12.3 g/dL) and serum ferritin levels (from a median of 3.4 ng/mL to 114.7 ng/mL).^32^

Limitations of this trial include those typical of similar clinical studies. The population was small and heterogeneous, which tempers the strength of the dose recommendation and any conclusions drawn from the pharmacodynamic data. The duration of follow-up was limited to 35 days, whereas the developmental effects of anemia can be much longer.^36^ Additionally, clinical symptoms of anemia beyond iron indices, such as fatigue, neurocognitive impairment, restless legs syndrome, and changes in hair quality,^37,38^ were not collected during the study.

The results of this study demonstrate that single FCM doses of 7.5 mg/kg and 15 mg/kg FCM can be safely administered intravenously and are well tolerated by pediatric patients aged 1 to 17 years. Dose-related increases in ferritin and TSAT and clinically meaningful increases in mean hemoglobin concentration were observed from baseline to the end of follow-up (Day 35). Improvements in iron indices and increases in hemoglobin were consistently greater with the higher dose of FCM (15 mg/kg) with similar safety profiles, which supports the use of the FCM 15 mg/kg in pediatric patients. FCM was recently approved by the US FDA at a dosage of 15 mg/kg for the treatment of IDA in pediatric patients and is an effective option for patients who are intolerant of or who have an unsatisfactory response to oral iron.

### Supplementary information

CONSORT Checklist

Supplemental Materials

## Data Availability

Summary data that support the findings of this study are available from the corresponding author upon reasonable request.
