# Supplementary material for: Safety, pharmacokinetics, and pharmacodynamics of intravenous ferric carboxymaltose in children with iron deficiency anemia
Source: Pediatr Res. 2023 May 19;94(4):1547–54. doi: 10.1038/s41390-023-02644-9 (PMC10589089; doi:10.1038/s41390-023-02644-9)
Supplement: Supplementary file 2 — Supplemental Materials [file 41390_2023_2644_MOESM2_ESM.pdf]

Supplemental Materials

Supplemental Figure 1. CONSORT Flow Diagram

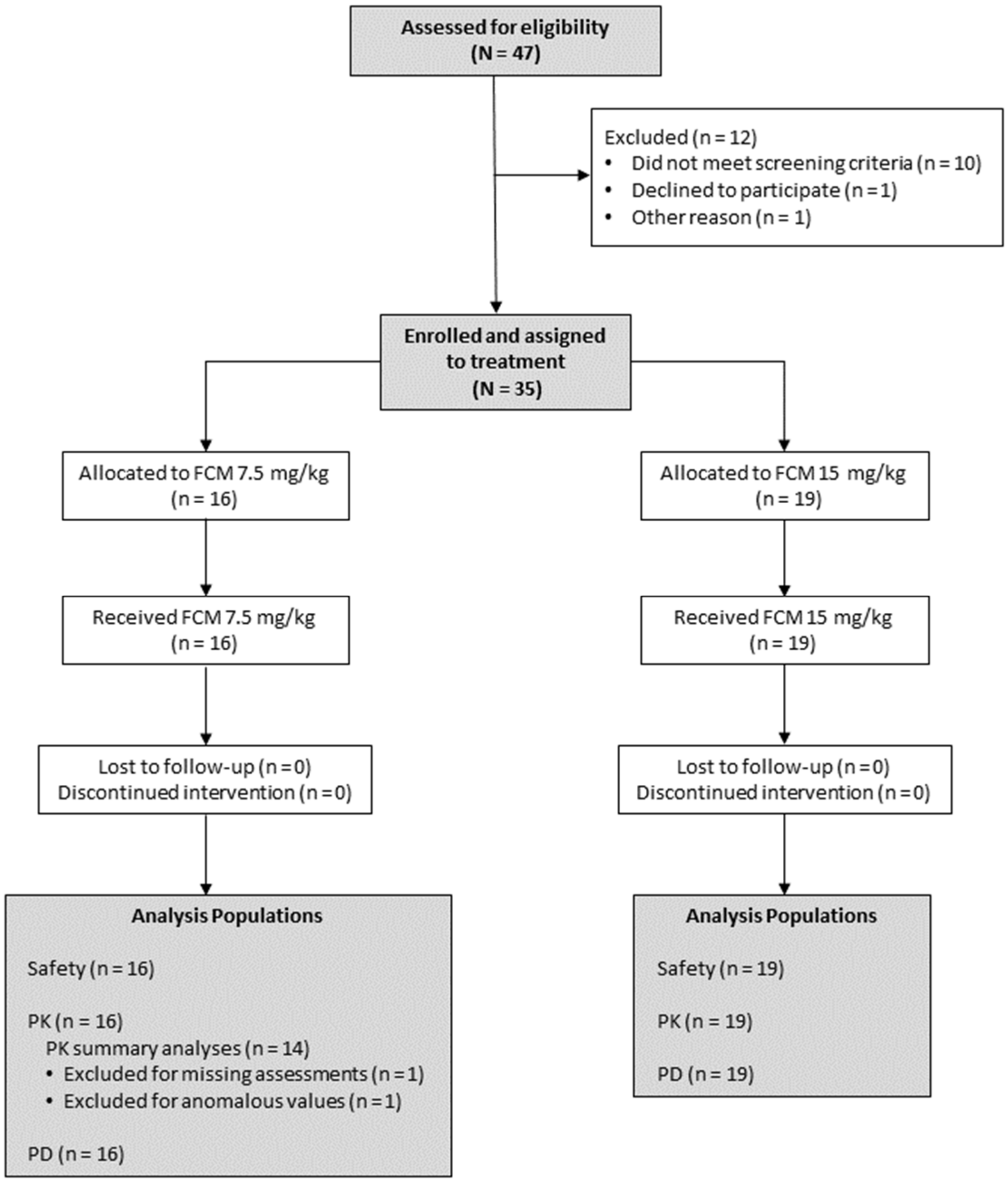

Supplemental Table 1. Phosphate levels over time in pediatric patients with iron deficiency anemia treated with FCM (safety population)

	FCM 7.5 mg/kg[a] (N = 16)				FCM 15 mg/kg[a] (N = 19)			
		Phosphate, mg/dL		Below LRL,		Phosphate, mg/dL		Below LRL,
Visit	N	Mean (SD)	Range	n (%)[b,c]	N	Mean (SD)	Range	n (%)[b,c]
Baseline	16	4.7 (0.70)	3.8 – 5.9	0 (0)	10	4.4 (0.59)	3.2 – 5.3	0 (0)
Day 3	16	4.4 (0.89)	2.9 – 5.6	1 (6.3)	7	3.0 (0.68)	2.0 – 4.0	2 (28.6)
Day 14	16	4.7 (0.64)	3.6 – 5.8	0 (0)	6	3.3 (1.02)	2.0 – 4.3	2 (33.3)
Day 28	16	5.0 (0.57)	4.1 – 5.8	0 (0)	4	4.3 (0.35)	3.9 – 4.7	0 (0)
Day 35	16	5.0 (0.52)	4.1 – 5.7	0 (0)	2	4.1 (0.28)	3.9 – 4.3	0 (0)

Abbreviations: FCM, ferric carboxymaltose; LRL, lower reference limit; SD, standard deviation.

[a] Maximum 750 mg.

[b] Normal phosphate ranges for female patients by age were as follows: 1–4 y: 3.4–6.0 mg/dL; 4–7 y: 3.2–5.5 mg/dL; 7–10 y: 3.1–5.5 mg/dL; 10–13 y: 3.3–5.3 mg/dL; 13–16 y: 2.8–4.8 mg/dL; 16–18 y: 2.5–4.8 mg/dL.

[c] Normal phosphate ranges for male patients by age were as follows: 1–4 y: 3.1–6.0 mg/dL; 4–7 y: 3.3–5.6 mg/dL; 7–10 y: 3.0–5.4 mg/dL; 10–13 y: 3.2–5.7 mg/dL; 13–16 y: 2.9–5.1 mg/dL; 16–18 y: 2.7–4.9 mg/dL.
